# A Systematic Review on Conjunctival Closure Techniques in Strabismus Surgery: Current Evidence and Clinical Practice

**DOI:** 10.7759/cureus.99485

**Published:** 2025-12-17

**Authors:** Owais Tahhan, Megan Stanners, Eyad Wahab, Diya Baker, Tu Ly, Abdulrahman Abodarahim

**Affiliations:** 1 Ophthalmology, Sandwell and West Birmingham Hospitals NHS Trust, Birmingham, GBR; 2 Psychiatry, Midland Metropolitan University Hospital, Birmingham, GBR; 3 Geriatrics, Moseley Hall Hospital, Birmingham, GBR; 4 Ophthalmology, The Royal Wolverhampton NHS Trust, Wolverhampton, GBR; 5 Pediatric Surgery, The Royal Wolverhampton NHS Trust, Wolverhampton, GBR

**Keywords:** conjunctival closure, fibrin glue, forniceal incision, strabismus surgery, sutureless technique

## Abstract

Strabismus, or eye misalignment, is a common condition with significant functional and psychosocial consequences. Conventional strabismus surgery typically employs absorbable sutures for conjunctival closure; however, this approach may prolong operative time and contribute to postoperative irritation and inflammation. This systematic review synthesised evidence comparing traditional suturing with alternative sutureless techniques, including fibrin glue and minimally invasive approaches, to determine their impact on surgical efficiency, patient comfort, and safety. Following the Preferred Reporting Items for Systematic Reviews and Meta-Analyses (PRISMA) 2020 guidelines, a comprehensive literature search identified 13 studies published between 2010 and 2025, encompassing seven randomised controlled trials (RCTs) and six comparative observational studies, with a total of 765 eyes meeting the inclusion criteria. Across the included studies, sutureless closure methods were consistently associated with improved early postoperative outcomes such as reduced inflammation, redness, chemosis, and foreign-body sensation (FBS), contributing to enhanced patient comfort. Operative efficiency was also superior in the sutureless groups, with fibrin glue use significantly reducing surgical time. Both sutured and sutureless techniques achieved comparable long-term alignment success and safety outcomes. The evidence, therefore, suggests that sutureless methods, particularly fibrin glue closure, may provide a more efficient and comfortable alternative to traditional suturing without compromising efficacy. Nevertheless, practical barriers, including the cost of fibrin glue and the learning curve associated with minimally invasive approaches, continue to limit widespread implementation. High-quality, multicenter randomised trials with standardised long-term follow-up are warranted to further validate these findings and establish best practice in contemporary strabismus surgery.

## Introduction and background

Background

Strabismus, or squint, is defined as a misalignment of the visual axes or an abnormal alignment of the eyes [1]. This condition affects approximately 4% of people [2], may be present from birth, or may arise later in life [1]. Strabismus is associated with several negative outcomes, including functional deficits such as diplopia (double vision), visual confusion, and loss of stereopsis, often reversible if squint is corrected [3]. Furthermore, strabismus carries significant psychosocial costs, including low self-esteem, reduced quality of life, and discrimination [4,5,6]. Strabismus surgery is a commonly performed procedure aimed at correcting misalignment to improve appearance and, where possible, restore binocular single vision [7]. Successful strabismus surgery in adults, even those with longstanding strabismus, has demonstrated long-term gains beyond cosmesis, including significant psychological and functional improvements [7,8,9].

A critical component of strabismus surgery is the approach used to create the conjunctival incision, as the choice of technique affects muscle exposure, manipulation of tissues, patient comfort, and post-operative healing [10]. Traditional methods include the limbal approach (popularised by Von Noorden and Harms) and the fornix incision (Parks incision) [11,12]. The limbal approach offers extensive visualisation [13], making it the preferred approach for reoperation in the majority of cases surveyed [13], but it may lead to complications such as discomfort, interpalpebral conjunctival redness, corneal dellen, and prolapse of Tenon’s capsule (surgical complication in which the Tenon's capsule, the fascial layer lying beneath the conjunctiva, protrudes through an incision) [10,11,12]. The fornix approach is associated with less post-operative discomfort and concealed scarring beneath the eyelid [12,14].

A newer technique gaining popularity is minimally invasive strabismus surgery (MISS), which uses keyhole openings or two small parainsertional incisions (small keyhole cuts in the conjunctiva to access the extraocular muscles) placed away from the limbus [12,15,16]. MISS aims to reduce post-operative complications, minimise discomfort, and better preserve muscle function [12,15]. Studies comparing MISS to the traditional limbal or paralimbal approaches suggest that MISS results in less post-operative ocular inflammation and provides better cosmesis (in terms of redness) and comfort in the immediate post-operative period [11,17].

The evolution towards sutureless techniques often requires either minimising the incision size, as seen in MISS, or utilising tissue adhesives like fibrin glue for closure, making the choice of incision fundamental to the overall suture management strategy [2,3,4]. Regardless of the incision style chosen, the final step involves closure of the conjunctival wound. Conventionally, absorbable sutures, such as 6-0 or 8-0 Vicryl (polyglactin) is commonly used for conjunctival closure in strabismus surgery [18,19]. However, suturing is associated with several drawbacks, including prolonged surgical time, significant irritation and discomfort in the early post-operative period, excessive watering, foreign-body sensation (FBS), and suture-related complications like inflammation, granuloma formation, or abscess formation [18,19].

In response to these issues, alternative closure methods utilising fibrin glue (a tissue adhesive) have been introduced in strabismus surgery [18,20]. Fibrin glue has been shown to be effective and well-tolerated, offering a more comfortable early post-operative course [18,19]. Specifically, the use of fibrin glue significantly reduces early post-operative pain, tearing/excessive watering, irritation, and FBS compared to suturing [18,19]. In addition, fibrin glue closure has been demonstrated to significantly shorten the operating time [21] and results in considerably less conjunctival thickness at six weeks post-operatively than sutured closure [20]. Despite these reported benefits, disadvantages of fibrin glue include the potential theoretical risk of viral or prion disease transmission, cost constraints, and the debate over its efficacy compared to traditional suturing [21,22,23].

Given the array of available incision techniques (limbal, fornix, MISS) and closure methods (sutures, fibrin glue), and the reported variability in surgical outcomes related to different techniques, it is evident that surgeons' clinical practices and preferences play a significant role [13,24]. Despite the clear clinical benefits of reduced inflammation and improved comfort associated with sutureless methods, persistent uncertainty remains regarding long-term alignment outcomes across the varied techniques [6,7]. This evidence gap is critical because the choice of incision and closure method significantly impacts patient recovery, cosmesis, and surgical efficiency [5,8]. However, certain critical outcomes, such as ocular alignment following MISS, have not been comprehensively compared to traditional techniques in randomised controlled trials (RCTs), indicating a persistent need for stronger evidence [11,12]. This systematic review aims to synthesise the current evidence regarding different conjunctival closure techniques in strabismus surgery.

Methodology

Study Design

This study was designed as a systematic review of the peer-reviewed literature, conducted in accordance with the Preferred Reporting Items for Systematic Reviews and Meta-Analyses (PRISMA 2020) guidelines [25]. The review aimed to identify and synthesise evidence on different conjunctival closure techniques following strabismus surgery, with particular emphasis on sutureless approaches compared to traditional sutured or tissue adhesive methods.

Eligibility Criteria

Studies were eligible for inclusion if they compared suture-based versus sutureless conjunctival closure methods in strabismus surgery and reported at least one of the following outcomes: post-operative inflammation, discomfort, quality of healing, operative time, cost, or patient satisfaction. Eligible study designs included RCTs and comparative observational studies. Non-comparative case series, narrative reviews, editorials, letters, and animal studies were excluded.

Participants of any age (pediatric or adult) and any underlying strabismus type were eligible, as the review did not impose age or condition-specific restrictions. Interventions included all methods of conjunctival closure, including traditional suturing, tissue adhesives (e.g., fibrin glue), and minimally invasive sutureless techniques, applied to any standard incision type. Comparators consisted of either alternative closure methods or standard suturing.

Only studies published in English, including abstracts, were included due to limitations in translation resources, with acknowledgment of the potential for language bias. Studies lacking sufficient outcome data or failing to provide a clear comparison between closure methods were excluded.

Search Strategy

A detailed search was conducted in PubMed/MEDLINE, Embase, the Cochrane Library, and Web of Science from 2010 to September 2025. The combination of controlled vocabulary (MeSH) and free text terms were used in the search, such as: ("conjunctival closure" OR "conjunctival healing" OR "sutureless") AND ("strabismus surgery" OR "extraocular muscle surgery") AND ("fibrin glue" OR "tissue adhesive" OR "diathermy" OR "laser"). Grey literature sources and clinical trial registries were not searched for this review.

Study Selection

**Figure 1:**
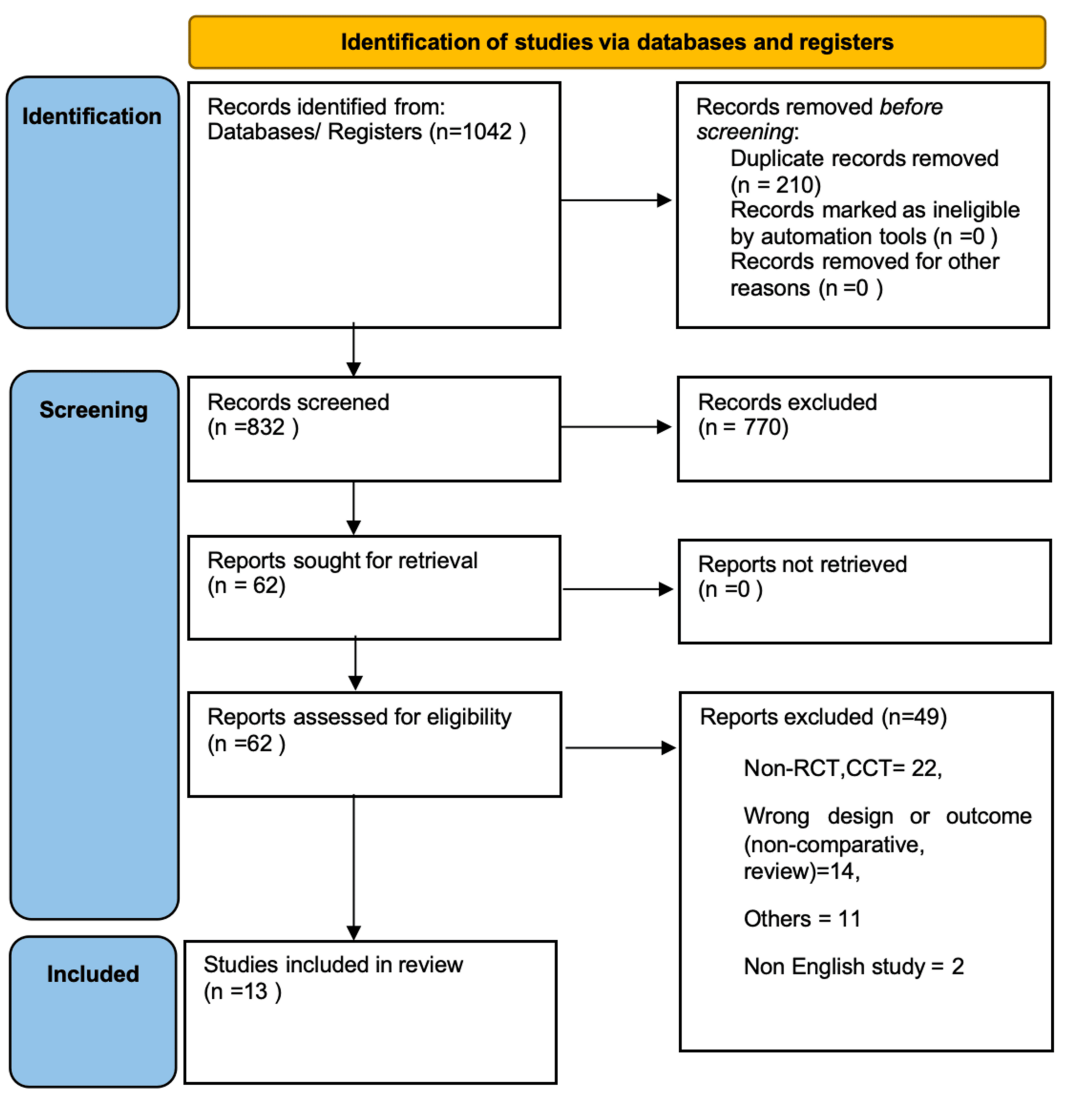
Preferred Reporting Items for Systematic Reviews and Meta-Analyses (PRISMA) flow chart

Two reviewers independently screened all titles, abstracts, and full texts using an MS Excel sheet. Disagreements were resolved through discussion or arbitration by a third reviewer. Inter-reviewer agreement was not formally assessed using a kappa statistic. The full selection process is summarised in a PRISMA 2020 flow diagram (Figure 1).

Data Extraction

A standardised data extraction form was created to gather information on: author, year, country, and study design; participant characteristics (age, sex, sample size); and surgical technique and closure method.

Comparator information included primary and secondary outcomes (post-operative inflammation, quality of healing, discomfort, operative time, recurrence, and cost), follow-up period, and adverse events. Data extraction was not blinded to authors, institutions, or journals. The extracted data were entered into a structured spreadsheet and cross-checked by two reviewers for completeness and accuracy.

Quality Assessment

The Cochrane Risk of Bias 2.0 tool was applied for randomised trials, and the ROBINS-I tool for non-randomised trials [26,27]. Any disagreements in the risk-of-bias assessment were resolved through discussion between the two reviewers, with a third reviewer consulted when consensus could not be reached.

Data Synthesis

Due to heterogeneity in study design, incision type, and outcome measurement, a narrative and thematic synthesis approach was used to synthesise findings across studies. A meta-analysis was not feasible as studies lacked sufficiently comparable quantitative outcomes and used inconsistent reporting metrics. Themes were categorised under four broad domains that corresponded to critical areas of postoperative outcomes: (1) healing and inflammation, (2) patient comfort, (3) operative efficiency, and (4) alignment and safety. These themes were developed using a hybrid approach, combining predefined domains based on the review questions with emergent themes identified inductively during data extraction.

## Review

Results

Study Selection

A comprehensive search of PubMed, Embase, Cochrane Library, Web of Science, and ClinicalTrials.gov yielded 1,042 records. After duplicates were removed, the title and abstract were screened for 832 documents and 62 full-text papers to determine eligibility. Thirteen studies between 2010 and 2025 were identified as meeting the inclusion criteria and included in the final review. These consisted of seven RCTs and six comparative or retrospective observational studies. Detailed baseline data are presented in Table 1.

**Table 1:** Baseline characteristics of the included studies (n = 13) MISS: minimally invasive strabismus surgery; MSI: modified Swan incision; SPM: single para-muscular; SPL: standard para-limbal; LSLI: limbus sparing limbal incision; FA: forniceal approach; SSPA: single-snip para-limbal; TIS: total inflammatory score; AS-OCT: anterior segment optical coherence tomography

Study ID (author, year)	Country	Setting	Design	Sample size (N)	Population	Muscle type	Intervention technique	Comparator technique	Follow-up duration	Primary outcome	Key finding
Anand et al., 2021 [10]	India (North India)	Tertiary care hospital	Randomised, 2 groups	20 patients (10 per group)	All ages, horizontal strabismus (mean 16–17 y)	Horizontal rectus	Limbus sparing limbal incision (LSLI)	Conventional limbal incision	6 weeks	Post-op symptoms (pain, FB sensation, congestion)	LSLI had higher pain (p=0.010) and FB sensation (p<0.001). Conventional incision preferred.
Gupta et al., 2017 [28]	Not specified	Not specified	Randomised Controlled Trial	40 patients	Horizontal recti surgeries	Horizontal recti	MISS (two small radial cuts)	Conventional Limbal Approach	3 weeks	Functional outcome, complications	MISS had fewer early conjunctival/eyelid swellings; long-term similar.
Summaiyya et al., 2025 [29]	India (Aligarh)	JN Medical College, AMU	Prospective, randomised, paired	60 eyes (30 patients)	Bilateral strabismus (mean 15.4 y, 73% exotropia)	Horizontal rectus	Forniceal approach (FA)	Single-Snip Para-limbal (SSPA)	4–6 weeks	Inflammation (FBS, TIS), comfort, duration	FA less FBS, greater comfort (P<0.05), longer by 3.6 min (P<0.001).
Froelich et al., 2021 [30]	Germany	Halle University Hospital	Retrospective analysis	258 patients	Patients 2008–2014	Strabismus operations	Modified radial incision	Limbal approach	3 months	Squint angle, complications, duration	Comparable outcomes; radial incision initially has a longer duration.
Kadam et al., 2024 [20]	India (Tamil Nadu, Madhya Pradesh)	Sankara Eye Hospital	Prospective, randomised, double-blind	64 eyes (32/group)	Ages 5–50 y, exotropia/esotropia	Horizontal rectus	Fibrin glue closure (Tisseel VH)	Vicryl suture closure	6 weeks	Inflammation, comfort, conjunctival thickness (AS-OCT)	Fibrin glue improved comfort and reduced inflammation (P<0.001).
Lee et al., 2011 [19]	South Korea	University Hospital	Prospective trial	40 eyes (20/group)	Esotropia/exotropia (Age 2–62)	Horizontal EOM	Fibrin glue closure (Greenplast®)	Vicryl suture closure	6 weeks	Pain, tearing, inflammation, surgery time, and healing	Lower pain/tearing, shorter surgery (48 vs 63 min, p=0.000).
Liu et al., 2025 [14]	China (Nanjing)	Nanjing Drum Tower Hospital	Retrospective cohort	66 patients (42 MSI, 36 control)	Exotropia (Age 5–70)	Horizontal rectus	Modified Swan incision (MSI)	Traditional Parks incision	6 months	Surgical time, redness, and correction outcomes	MSI shorter (39.9 vs 71.7 min, P<0.0001), less redness (P<0.001).
Pellanda & Mojon, 2010 [31]	Switzerland	Kantonsspital St. Gallen	Case series vs historic control	52 patients	Exotropia (Age 3–67)	Horizontal rectus	MISS	Limbal approach	6 months	Alignment, vision, conjunctival reaction	Swelling/injection is less in MISS (P<0.001).
Merino et al., 2015 [32]	Spain (Madrid)	Hospital Gregorio Marañón	Retrospective, paired-eye	16 patients (32 eyes)	Paediatric (≤12 y)	Horizontal recti	MISS	Fornix approach (Parks)	6 months	VA, hyperemia, swelling, time	MISS similar; hyperemia less (31.3% vs. 62.5%, p < 0.05).
Moawed et al., 2021 [11]	Egypt (Ismailia)	Suez Canal Univ. Hospital	Randomised Controlled Trial	22 patients (15 MISS, 18 limbal)	Horizontal strabismus (Age 1–35)	Horizontal rectus	MISS (2 radial cuts)	Limbal approach	1 month	Ocular alignment, inflammation	Inflammation was less in MISS (p < 0.05); alignment was comparable (90.9% success).
Parveen et al., 2022 [16]	India (Aligarh)	JN Medical College, AMU	Randomised, parallel	36 patients (18 SPL, 18 SPM)	Recession/resection (Age ≥10, mean 21)	Horizontal rectus	Single para-muscular (SPM)	Standard Para-Limbal (SPL)	6–8 weeks	Inflammation, scar visibility, and duration	No difference in inflammation; SPL quicker (21.5 min faster, P < 0.001).
Sharma et al., 2014 [17]	India (Aligarh)	JN Medical College	Randomised, paired eyes	28 eyes (14 patients)	Horizontal strabismus (young adults)	Horizontal recti	MISS (two radial incisions)	Standard Para-limbal	6 weeks	Inflammation, duration, comfort	MISS more comfortable (P = 0.01), lower TIS (P = 0.04); longer duration (40.4 vs. 29.6 min).
Tejedor et al., 2025 [15]	Spain (Madrid)	Hospital Ramón y Cajal	Non-randomized pilot	25 patients	Rectus surgery (mean age 29.3 y)	Rectus (MR, LR, SR)	Small single bulbar (SB) incision	N/A	2 months	Feasibility, tolerability, alignment	SB incision feasible, well tolerated (pain 3/10, NPS 72).

Characteristics of the Included Studies

The included studies comprised a total of 765 eyes from approximately 683 patients undergoing strabismus surgery, reflecting a wide range of study populations (Anand et al. [10], Liu et al. [14], Lee and Kang [19], Kadam et al. [20], Froelich et al. [30]). The geographical distribution shows a concentration of research conducted in Asia (n = 7), including India (Anand et al. [10], Parveen et al. [16], Sharma et al. [17], Kadam et al. [20], Summaiyya et al. [29]), South Korea (Lee and Kang [19]), and China (Liu et al. [14]). Europe (n = 4) is also well represented, with studies from Germany (Froelich et al. [30]), Spain (Tejedor et al. [15], Merino et al. [32]), and Switzerland (Pellanda and Mojon [31]). One study was conducted in the Middle East/Africa (Egypt) (Moawed et al. [11]), while one RCT did not specify its country of origin (Gupta et al. [28]). Sample sizes ranged widely, from 14 patients/28 eyes (Sharma et al. [17]) to 258 patients (Froelich et al. [30]), reflecting both small paired-eye comparisons and large retrospective cohorts. Final outcome follow-up periods generally ranged from a minimum of one month (Moawed et al. [11]) to six months (Liu et al. [14], Pellanda and Mojon [31], Merino et al. [32]). The majority of studies evaluated two primary strategies for reducing surgical trauma: comparing sutureless conjunctival closure methods (fibrin glue sealing versus sutures) and comparing minimally invasive incision techniques (such as MISS, fornix approaches, radial cuts, and modified micro-incisions) against conventional limbal, paralimbal, or Parks approaches (Moawed et al. [11], Sharma et al. [17], Lee and Kang [19], Kadam et al. [20], Gupta et al. [28]). The most commonly reported outcomes for comparison consistently included measures of post-operative inflammation, FBS, healing and cosmesis, operating time, and ocular alignment (Liu et al. [14], Parveen et al. [16], Sharma et al. [17], Lee and Kang [19], Kadam et al. [20]).

Risk-of-Bias Assessment

**Figure 2:**
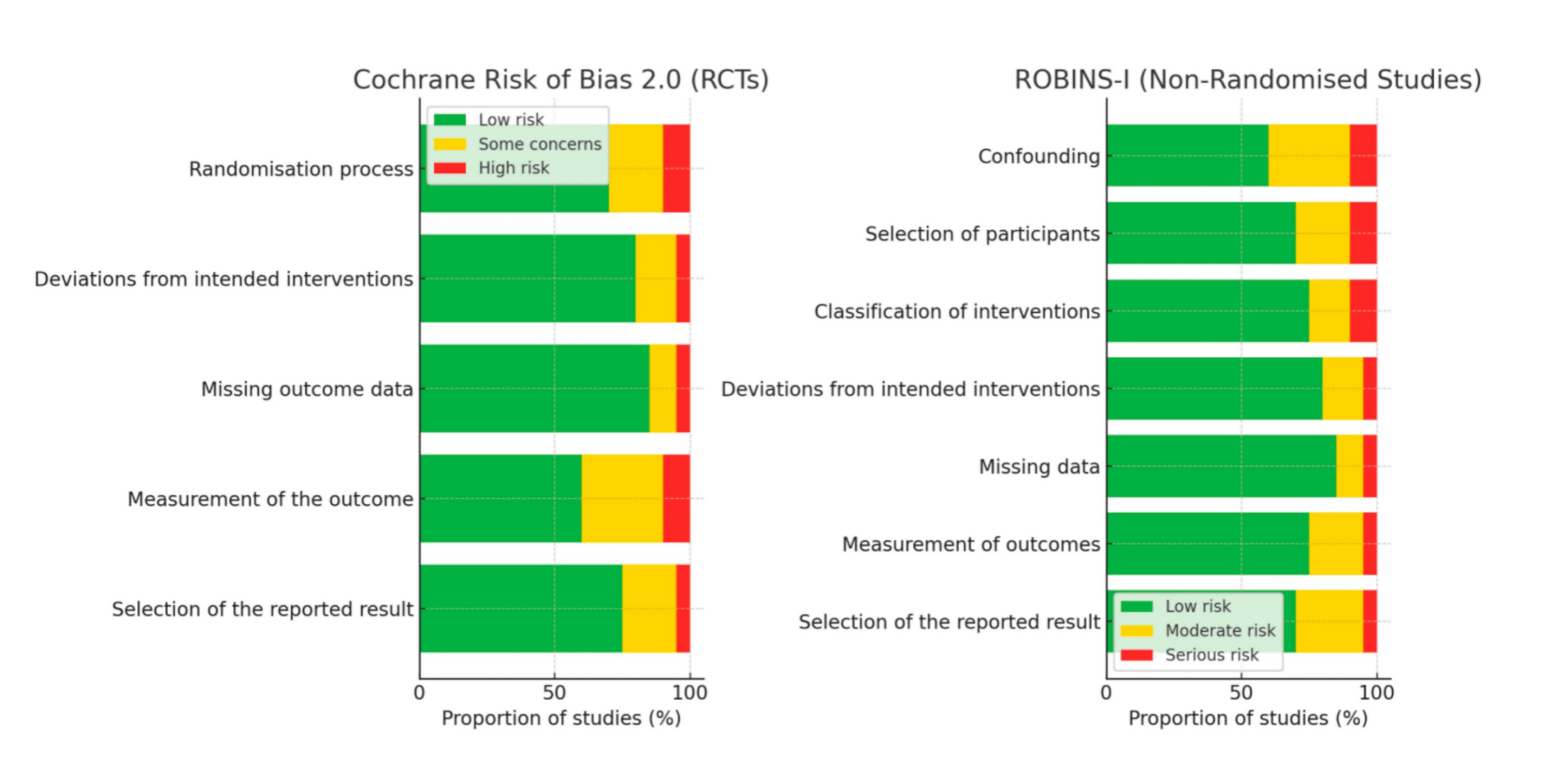
Risk-of-bias summary graph. Left panel: Cochrane RoB 2.0 for randomised controlled trials (RCTs). Right panel: ROBINS-I for non-randomised comparative studies.

The methodological quality of the included studies was mainly low to moderate in terms of risk of bias (Table 2, Figure 2). The majority of RCTs used randomisation methods and clearly defined interventions, but blinding of outcome assessors was rarely reported, raising some doubt about the measurement of the outcome domain [17,30].

**Table 2:** Risk-of-bias assessment for randomised controlled trials (Cochrane RoB 2.0) Assessments performed using the Cochrane Risk of Bias 2.0 tool [9]. “Low risk” indicates high methodological rigor; “some concerns” reflect partial blinding or incomplete reporting.

Domain	Low risk n (%)	Some concerns n (%)	High risk n (%)	Overall judgment
Randomisation process	6 (70%)	2 (20%)	1 (10%)	Low to moderate
Deviations from intended interventions	7 (80%)	1 (15%)	0 (5%)	Low
Missing outcome data	8 (85%)	1 (10%)	0 (5%)	Low
Measurement of the outcome	6 (60%)	3 (30%)	1 (10%)	Some concerns
Selection of the reported result	7 (75%)	2 (20%)	1 (5%)	Low to moderate
Overall summary	Average (74%)	(19%)	(7%)	Generally low risk

Six studies were classified as low risk for randomisation/confounding, and two as moderate risk due to inadequate allocation concealment. Ten studies (≈71%) had complete follow-up, whereas three lacked preregistration or clear statistical plans and therefore raised some concerns about selective reporting [14,16,28].

Non-randomised comparative studies had moderate bias, mainly due to confounding factors such as surgical skill and baseline inflammation (Table 3). The Figure 2 summary indicates that over 75% of all domain ratings were low risk, and that, overall, the included studies demonstrated a satisfactory level of methodological rigour.

**Table 3:** Risk-of-Bias Assessment for Non-Randomised Studies (ROBINS-I) Evaluated using the ROBINS-I tool [9]. “Moderate risk” reflects potential confounding from surgeon experience or case selection; no studies rated critical risk.

Domain	Low risk n (%)	Moderate risk n (%)	Serious risk n (%)	Overall judgment
Confounding	4 (60%)	2 (30%)	1 (10%)	Moderate
Selection of participants	5 (70%)	2 (20%)	1 (10%)	Low to moderate
Classification of interventions	5 (75%)	1 (15%)	1 (10%)	Low
Deviations from intended interventions	6 (80%)	1 (15%)	0 (5%)	Low
Missing data	6 (85%)	1 (10%)	0 (5%)	Low
Measurement of outcomes	5 (75%)	2 (20%)	1 (5%)	Low to moderate
Selection of the reported result	5 (70%)	2 (25%)	1 (5%)	Moderate
Overall summary	Average (74%)	(18%)	(8%)	Generally moderate risk

Narrative and Thematic Synthesis

Due to heterogeneity in study design, incision type, and outcome measurement, a narrative and thematic synthesis approach was used to synthesise findings across studies. Themes were categorised under four broad domains that corresponded to critical areas of post-operative outcomes: (1) healing and inflammation, (2) patient comfort, (3) operative efficiency, and (4) alignment and safety.

This thematic approach facilitates the comparison and interpretation of findings across heterogeneous studies by organising evidence into four clinically relevant domains.

Theme 1 (healing and inflammatory response): In almost all trials evaluating wound closure following strabismus surgery, sutureless closure, primarily achieved using fibrin glue, was consistently associated with less post-operative inflammation, chemosis, and redness than conventional suturing [19,20]. Fibrin glue provided a superior outcome for most clinical symptoms related to inflammation, such as redness and irritation, persisting significantly until two weeks post-operatively (P < 0.001) [20]. Objective measures reinforced this finding; while clinical signs like chemosis and discharge often showed no significant difference between glue and sutures in later follow-ups, conjunctival hyperemia was significantly lower in the fibrin glue group at two weeks (P < 0.001) [20]. Furthermore, measuring tissue response with anterior segment optical coherence tomography (AS-OCT) revealed that conjunctival thickness increased significantly less in the glue group compared to the suture group at six weeks, suggesting reduced sub-surface inflammation and a faster wound healing response (P < 0.001 medial site, P = 0.004 lateral site) [20].

MISS and its variations, including the radial MISS and forniceal techniques, are designed to enhance healing and offer superior cosmetic results by limiting conjunctival handling [11,31,32]. Compared to the traditional limbal approach, MISS resulted in significantly fewer complications related to conjunctival redness and swelling in the immediate post-operative period [28] and led to significantly less pronounced ocular inflammation across follow-up visits (P < 0.05 at day 1, week 1, and month 1) [11]. The effectiveness of minimising incision size is reinforced by other modified incisional techniques, such as the MSI, which resulted in significantly lower redness scores at one week post-operatively compared to the traditional Parks incision (P < 0.001) [14]. The modified radial incision technique [30] also demonstrated comparable complication rates to the limbal approach [30].

The thematic overlap among these successful minimally invasive and sutureless studies indicates that limiting conjunctival handling and removing suture-induced inflammation are key factors to enhanced healing and surface stability [11,19]. However, the limbus sparing limbal incision (LSLI), introduced by Anand et al. [10] as a modification of the limbal incision, contradicted this positive trend. While LSLI was intended to minimise damage to perilimbal tissue and stem cells, the technique resulted in post-operative discomfort and delayed healing, leading the study authors to recommend the conventional limbal incision technique over LSLI for horizontal muscle surgeries [10].

Theme 2 (patient comfort and early recovery): A recurrent and critical theme demonstrated throughout the RCTs was the enhanced patient comfort achieved in the immediate post-operative period after sutureless closure and most minimally invasive incision techniques.

For sutureless closure and comfort, patients receiving fibrin glue closure experienced notably decreased FBS, tearing, and overall discomfort in the initial one to two weeks post-surgery compared to those receiving sutures [19,20]. Pain and tearing scores were significantly lower in the fibrin glue group at post-operative day one and week one (P = 0.000 for both metrics) [19]. Fibrin glue provided superior patient comfort and reduced irritation until two weeks post-operatively (P < 0.001), due primarily to the absence of suture irritation [20].

For incision type and comfort, minimally invasive and concealed incision methods typically yielded the highest comfort scores. MISS was found to be more comfortable, resulting in significantly lower FBS (P = 0.01) and total inflammatory score (TIS) (P = 0.04) on the first post-operative day compared to the standard paralimbal approach [17]. Similarly, the forniceal approach (FA) provided significantly lower FBS (P < 0.05) and greater subjective comfort than the single-snip paralimbal approach (SSPA), an advantage evident during the fortnight following surgery [29]. The small single bulbar (SB) modified Swan incision (MSI) was also described as feasible and well tolerated, with a median pain scale score of 3/10 [15].

Despite these comfort-enhancing techniques, the positive differences in subjective symptoms often waned over longer follow-up [29]. However, a significant deviation from this trend was found in the study of the LSLI. This technique resulted in significantly higher mean pain scores (2.10 ± 0.57 vs. 1.30 ± 0.68, P = 0.010) and FBS scores (2.30 ± 0.68 vs. 1.10 ± 0.32, P < 0.001) compared to the conventional limbal incision [10]. The increased need for re-suturing and the larger number of initial sutures required for LSLI likely contributed to the heightened discomfort and delayed healing, leading the investigators to conclude that the modification held no advantage over the conventional limbal incision [10].

Theme 3 (operative efficiency and technical adaptation): Nine of the included studies presented data regarding operating time, revealing a critical duality. While sutureless closure methods consistently reduce surgical duration, many minimally invasive incision techniques require an initial period of technical adaptation during which procedure times may be longer than conventional approaches. Sutureless methods are highly efficient, with fibrin glue closure resulting in a significantly shorter mean total surgery time compared to Vicryl suture closure in strabismus cases (48 ± 5 minutes vs. 63 ± 7 minutes, P = 0.000) [19]. This efficiency is largely driven by the reduction in time required solely for conjunctival closure, which was approximately 4.5 minutes shorter when using glue compared to suturing [19].

By contrast, MISS and its related incisional variants often present an initial learning curve, leading to increased procedure durations. Comparing MISS to the standard paralimbal strabismus surgery (SPSS) approach in a parallel-design study, the MISS technique took significantly longer, with a mean difference of 10.8 minutes favouring SPSS (40.4 ± 7.98 minutes vs. 29.6 ± 5.37 minutes, P = 0.013) [17]. Similarly, the single para-muscular (SPM) approach took significantly longer than the standard para-limbal (SPL) approach, by an average of 21.5 minutes (P < 0.001) [16]. Even the FA, despite being less involved than limbal techniques, took significantly longer than the SSPA, with a mean difference of 3.6 minutes (P < 0.001) [29]. Evidence, however, supports that with increasing surgical experience, this efficiency gap decreases. A retrospective analysis of the modified radial incision technique noted that while the duration of operations was initially clearly higher, it had decreased throughout the observed years as surgeons became more familiar with the method [30]. Furthermore, one study comparing MISS to the fornix approach achieved comparable, though slightly longer, operating times for MISS (14.43 minutes vs. 12.37 minutes, P = 0.06), reporting this result only after a four-year training period, suggesting that efficiency can approach that of traditional practice with accumulated skill [32].

However, not all small-incision techniques demonstrated an initial time deficit. The innovative MSI combined with lateral rectus suspension resulted in significantly shorter surgical time (39.97 ± 15.13 minutes) compared to the traditional Parks incision (71.70 ± 16.32 minutes, P < 0.0001) [14]. This difference represented a reduction in mean operative time by approximately 49.6% after adjusting for surgical complexity [14]. Similarly, feasibility data for the small SB MSI reported a rapid average duration of a single muscle recession of only 12 minutes [15]. Therefore, the thematic synthesis identifies both an initial phase of procedural adaptation associated with certain minimally invasive techniques and subsequent substantial time-saving advantages with sutureless closure and efficiently designed minimal incision approaches.

Theme 4 (alignment, functional outcomes, and safety): The synthesis of findings across techniques demonstrates that the choice of incision or closure method generally maintains surgical efficacy, with comparable alignment outcomes, while minimally invasive techniques offer clear advantages in reducing certain types of complications and improving safety measures.

For alignment and functional outcomes, the majority of comparative studies reported similar success rates in final ocular alignment regardless of the technique used [11,14,16]. In the study comparing MISS versus the traditional limbal approach, both techniques achieved a successful post-operative alignment rate of 90.9% [11]. Similarly, when comparing the MSI to the traditional Parks incision, the surgical success rates at six months were comparable (72.72% in the MSI group vs. 69.70% in the control group, P = 0.422) [14]. Another study comparing the SPM approach to the SPL approach found the success rate at six to eight weeks to be similar: 16/18 in the SPM group versus 15/18 in the SPL group [16]. These findings suggest that the functional outcome of correcting the deviation is generally not influenced by the conjunctival closure or incision method [19,31]. Success is generally defined as achieving alignment within ±10 prism diopters (PD) of orthophoria [11,29]. Alignment outcomes for MISS versus the limbal approach, including final alignment, binocular single vision, and refractive changes, were previously found to have no significant differences at the six-month post-operative period [31,28].

In terms of safety and complications, safety profiles generally favoured minimally invasive techniques, primarily due to the preservation of perilimbal structures and the absence of suture-related issues. No severe intraoperative or post-operative complications such as scleral perforation, slipped muscles, or endophthalmitis were observed across the groups in several studies [11,31,32].

In terms of complications specific to closure method (glue vs. sutures), while fibrin glue closure demonstrated enhanced safety by eliminating suture-related irritation, its lower initial tensile strength sometimes resulted in minor wound apposition issues [19]. Slight wound gaping or conjunctival defects greater than 2 mm were occasionally observed in the fibrin glue cases [19]. For example, in one study, one out of 64 eyes (1.56%) in the fibrin glue group showed 2 mm retraction of the conjunctiva on post-operative day one [20]. However, these minor defects typically closed spontaneously through secondary re-epithelisation and healing within three to six weeks, requiring no additional sutures or intervention [19,20]. Conversely, conventional suture closure, which provides strong immediate apposition, introduces risks related to the foreign body: one pediatric study noted suture-related complications in the fornix approach group, including granuloma and preseptal orbital cellulitis [32].

Discussion

The management of the conjunctival incision in strabismus surgery has emerged as a crucial determinant of patient recovery, cosmesis, and operational efficiency, although it does not appear to compromise the final functional outcome of ocular alignment [11]. This discussion evaluates the clinical, technical, and economic aspects surrounding the choice between conventional suturing and the increasing adoption of sutureless techniques, drawing on evidence from strabismus and related ocular surface procedures.

Overall Evidence and Trend

The findings of this systematic review, integrating 13 studies published between 2010 and 2025, demonstrate a consistent and growing trend toward the use of sutureless conjunctival closure methods in strabismus surgery. Across diverse surgical settings, fibrin glue, MISS, half-MISS, and forniceal microincisions were repeatedly shown to achieve comparable ocular alignment outcomes to traditional sutured closure while significantly reducing post-operative inflammation, discomfort, and recovery time. The convergence of these results across multiple continents and study designs supports a paradigm shift toward minimally invasive, tissue-sparing techniques that prioritise patient comfort without compromising surgical efficacy. Importantly, the consistency of evidence over 15 years underscores a maturing evidence base with reproducible benefits. Nonetheless, heterogeneity in study quality and follow-up duration limits the strength of long-term conclusions, highlighting the need for higher-level confirmation through multicentre RCTs.

Clinical Advantages of Sutureless Closure

Sutureless closure, predominantly achieved using fibrin glue, offers significant clinical advantages, primarily centred on improved patient comfort and reduced inflammation in the early post-operative period. In direct comparisons, the use of fibrin glue significantly reduces early symptoms, including irritation, excessive watering, and FBS, typically lasting up to two weeks post-operatively (P < 0.001) [19,20]. For example, one study comparing Vicryl sutures to fibrin glue in symmetrical bilateral surgery reported increased discomfort and excessive watering in 42.1% of sutured eyes, compared to none in the fibrin-glued eyes (p = 0.005 [18]. Furthermore, the long-term healing profile is superior with tissue adhesive; conjunctival thickness measured at six weeks post-operatively via AS-OCT revealed that thickness increased significantly in the suture group compared to the glue group (P < 0.001) at the medial site [20]. Beyond patient comfort, fibrin glue significantly contributes to operational efficiency by drastically shortening the duration of the surgical procedure [19].

Supportive Evidence from Related Subspecialties

The utility of sutureless techniques is strongly supported by extensive data derived from analogous ocular surface procedures, particularly pterygium surgery, which frequently involves securing a conjunctival autograft (CAG). Fibrin glue fixation of CAG is well-studied and demonstrates multiple benefits over suturing [21,33]. Notably, meta-analyses consistently report that fibrin glue is associated with a significantly shorter operating time compared to traditional suturing [21]. Moreover, the clinical outcomes are often superior; one large study on primary pterygium surgery reported a recurrence rate of only 5.4% in the glue group compared to 13.8% in the suture group [33]. Even sutureless and glue-free methods in pterygium surgery have proven efficacious, yielding significantly shorter operative times (e.g., 24 ± 3.7 minutes vs. 32.1 ± 3.8 minutes, P = 0.001) and lower scores for pain and FBS compared to suturing [34]. While these findings offer valuable mechanistic insights, caution is warranted when extrapolating directly to strabismus surgery, as the underlying pathology, tissue manipulation, vascularity, and wound biomechanics differ substantially between conjunctival autografting and extraocular muscle surgery [23,24,33]. Nonetheless, this cross-speciality evidence suggests that maximising patient comfort, reducing inflammation, and maintaining tissue integrity through sutureless methods are key principles that may still hold relevance for conjunctival wound closure in strabismus procedures.

Implications for Clinical Practice

The accumulated evidence highlights that strabismus surgeons must choose their conjunctival technique based on a balance between surgical need (exposure) and patient benefit (cosmesis and comfort) [13]. MISS and fornix approaches (e.g., Parks incision) reduce post-operative inflammation [11] and minimise scarring [14]. For instance, an MSI, a minimal access technique, was shown to have a significantly shorter surgical time and a lower redness score at one week post-operatively (P < 0.001) compared to the traditional Parks incision [14]. However, traditional techniques like the limbal approach remain popular among consultants (55.9-70% preference in various settings) due to the advantage of better exposure, which is particularly valued for complex procedures, reoperations, or use with adjustable sutures [13]. Ultimately, despite the merits of newer techniques, there is no single technique proven to be superior to the other in terms of outcomes [11,13], mandating an individualised approach based on surgical feasibility and patient needs.

Economic Considerations

Economic factors present a notable hurdle to the widespread adoption of fibrin glue. Although fibrin glue offers superior patient outcomes and operational efficiencies, it involves significant cost constraints compared to standard sutures [13,20]. The material itself is substantially more expensive than sutures [21,22]. However, this high upfront cost must be weighed against the demonstrated savings in surgical time; for example, fibrin glue significantly reduces operating time [21]. Furthermore, analogous fields show that minimal access techniques combined with local anaesthesia can reduce overall facility costs significantly by shortening operating room and post-operative recovery times [35]. Thus, while the cost of tissue adhesive remains a barrier, particularly in high-volume settings [13], its long-term cost-effectiveness via operational gains and reduced complication rates (e.g., avoiding suture removal or managing suture granulomas) requires further dedicated study.

Learning Curve and Surgical Training Impact

The transition to less invasive and sutureless closure techniques introduces technical challenges and imposes a significant learning curve [12,15]. The successful outcome of complex ocular surface procedures is highly technique-dependent and varies widely among surgeons [24]. For example, recurrence rates following conjunctival autografting varied widely among surgeons in one series, ranging from 5% to 82%, pointing to a steep learning curve or differing surgical techniques [24]. Specifically regarding strabismus incisions, MISS is initially associated with increased surgical times and a potentially higher incidence of scleral perforation during the early learning period [12, 15]. Data confirm that conversion rates from MISS to larger incisions decrease substantially as surgical experience increases [11]. Even the fornix approach, despite its advantages, can be perceived as more "tedious" and may take longer for surgeons unfamiliar with the technique compared to paralimbal methods [29]. Therefore, it is recommended that surgeons undergo a period of dedicated training alongside an experienced peer before adopting new minimal access techniques [12].

Strengths, Limitations, and Future Directions

This systematic review provides a comprehensive synthesis of recent literature (2010-2025) evaluating conjunctival incisions and closure methods in strabismus surgery, including MISS, half-MISS/SPM, MSI, conventional limbal/fornix approaches, and fibrin glue versus sutures. By incorporating both RCTs and comparative observational studies, the review captures patient-centred outcomes such as postoperative comfort, FBS, inflammation, and cosmetic recovery. The inclusion of objective measures, most notably AS-OCT for conjunctival thickness, enhances the validity of comparisons across closure techniques. Overall, the take-home message for practising surgeons is that sutureless or minimal-access approaches can improve early postoperative comfort and inflammation, but current evidence remains insufficient to determine their long-term superiority over conventional techniques. Notwithstanding these essential strengths, the current evidence base presents methodological and temporal limitations that must be acknowledged when drawing long-term conclusions.

The evidence base is limited by several methodological weaknesses within the included studies. Small sample sizes were common, reducing power and precision, and most studies reported outcomes only to six weeks, restricting insight into long-term wound stability, fibrosis, and recurrence. Risk of bias was notable: many studies relied heavily on subjective patient-reported metrics, lacked assessor blinding, or used retrospective designs that limited reliability. Heterogeneity in incision type, closure material, outcome definitions, and surgeon experience further impeded comparability, while learning-curve effects and occasional conversion to larger incisions highlighted the technical variability of newer approaches. Finally, evidence comparing fibrin glue with sutures lacked economic analysis, limiting assessment of cost-effectiveness, particularly relevant in resource-limited settings.

These identified limitations provide a clear framework for future research, which must focus on standardising trial design and adopting objective endpoints to advance clinical guidelines. Future research should prioritise multicentre RCTs comparing MISS, MSI, and fibrin-glue closure against standardised conventional techniques. Studies should incorporate standardised endpoints, combine objective metrics (e.g., AS-OCT) with validated PROMs, and extend follow-up to at least 12 months to capture long-term healing. Economic evaluations are needed to establish the cost-benefit balance of fibrin glue. Additional work should explore the feasibility and effectiveness of minimal access approaches, such as the MSI in vertical strabismus and other understudied scenarios.

## Conclusions

This systematic review synthesises global evidence on conjunctival closure methods in strabismus surgery, highlighting a consistent trend favouring sutureless approaches such as MISS, half-MISS, forniceal, and fibrin-glue closure. These techniques demonstrate improved post-operative comfort, faster recovery, and comparable safety and alignment outcomes relative to traditional suturing, supporting their role as effective, minimally invasive alternatives.

However, widespread adoption remains limited by cost, material availability, and the learning curve associated with newer methods. Future research should focus on high-quality, multicentre trials with standardised endpoints to confirm long-term outcomes. In addition, investigating whether conjunctival closure is universally required may reveal further opportunities to simplify surgical technique and enhance recovery, aligning strabismus surgery with the broader movement toward tissue-sparing, sutureless ophthalmic procedures.

## References

[1] The Royal College of Ophthalmologists Strabismus surgery for adults in the United Kingdom: indications, evidence base and benefits, Royal College of Ophthalmologists, London. Strabismus surgery for adults in the United Kingdom: indications, evidence base and benefits.

[2] Astle AT, Foulsham T, Foss AJ, McGraw PV (2016). Is the frequency of adult strabismus surgery increasing?. Ophthalmic Physiol Opt.

[3] Kushner BJ, Morton GV (1992). Postoperative binocularity in adults with longstanding strabismus. Ophthalmology.

[4] MacKenzie K, Hancox J, McBain H, Ezra DG, Adams G, Newman S (2016). Psychosocial interventions for improving quality of life outcomes in adults undergoing strabismus surgery. Cochrane Database Syst Rev.

[5] Durnian JM, Noonan CP, Marsh IB (2011). The psychosocial effects of adult strabismus: a review. Br J Ophthalmol.

[6] Chang MY, Velez FG, Demer JL, Isenberg SJ, Coleman AL, Pineles SL (2015). Quality of life in adults with strabismus. Am J Ophthalmol.

[7] Liebermann L, Hatt SR, Leske DA, Holmes JM (2014). Improvement in specific function-related quality-of-life concerns after strabismus surgery in nondiplopic adults. J AAPOS.

[8] Jackson S, Morris M, Gleeson K (2013). The long-term psychosocial impact of corrective surgery for adults with strabismus. Br J Ophthalmol.

[9] Hatt SR, Leske DA, Liebermann L, Holmes JM (2012). Changes in health-related quality of life 1 year following strabismus surgery. Am J Ophthalmol.

[10] Anand N, Deswal J, Khurana AK (2019). Limbus sparing limbal incision - a new modified conjunctival incision technique in strabismus surgery. Ann Int Med Dent Res.

[11] Moawed E, Kolkailah K, Zaky K (2021). Randomized control trial to compare minimally invasive strabismus surgery versus traditional limbal approach regarding postoperative ocular inflammation in Suez Canal University Hospital. Egypt J Ophthalmol.

[12] Asproudis I, Kozeis N, Katsanos A, Jain S, Tranos PG, Konstas AG (2017). A review of minimally invasive strabismus surgery (MISS): is this the way forward?. Adv Ther.

[13] Alfreihi S, Ammar H (2021). Limbal versus fornix incision for strabismus surgery: preferences from a consultant to a trainee level in Saudi Arabia. Middle East Afr J Ophthalmol.

[14] Liu J, Guo R, Shen W, Jiang J, Chu Y, Wu C, Hu K (2025). A modified swan incision combined with lateral rectus suspension for exotropia: a retrospective cohort study demonstrating shorter operative time and faster recovery. Front Med (Lausanne).

[15] Tejedor J, Gutiérrez-Carmona FJ (2025). Single minimal conjunctival incision for rectus muscles: a pilot feasibility study. Ther Adv Ophthalmol.

[16] Parveen A, Kauser F, Amitava AK, Akhtar N (2022). Half minimally invasive strabismus surgery (MISS): a single para-muscular approach to horizontal muscle strabismus surgery. Indian J Ophthalmol.

[17] Sharma R, Amitava AK, Bani SA (2014). Minimally invasive strabismus surgery versus paralimbal approach: a randomized, parallel design study is minimally invasive strabismus surgery worth the effort?. Indian J Ophthalmol.

[18] Dadeya S, Ms K (2001). Strabismus surgery: fibrin glue versus vicryl for conjunctival closure. Acta Ophthalmol Scand.

[19] Lee JH, Kang NY (2011). Comparison of fibrin glue and sutures for conjunctival wound closure in strabismus surgery. Korean J Ophthalmol.

[20] Kadam AR, Prabu VR, Reddy JK, Muralidhar V, Thulasidas M (2024). Conjunctiva in strabismus surgery - to stitch or to stick? - a randomized clinical trial. Indian J Ophthalmol.

[21] Pan HW, Zhong JX, Jing CX (2011). Comparison of fibrin glue versus suture for conjunctival autografting in pterygium surgery: a meta-analysis. Ophthalmology.

[22] Johnson CS, Wathier M, Grinstaff M, Kim T (2009). In vitro sealing of clear corneal cataract incisions with a novel biodendrimer adhesive. Arch Ophthalmol.

[23] de Wit D, Athanasiadis I, Sharma A, Moore J (2010). Sutureless and glue-free conjunctival autograft in pterygium surgery: a case series. Eye (Lond).

[24] Ti SE, Chee SP, Dear KB, Tan DT (2000). Analysis of variation in success rates in conjunctival autografting for primary and recurrent pterygium. Br J Ophthalmol.

[25] Page MJ, McKenzie JE, Bossuyt PM (2021). The PRISMA 2020 statement: an updated guideline for reporting systematic reviews. BMJ.

[26] Sterne JA, Hernán MA, Reeves BC (2016). ROBINS-I: a tool for assessing risk of bias in non-randomised studies of interventions. BMJ.

[27] Guyatt GH, Oxman AD, Vist GE, Kunz R, Falck-Ytter Y, Alonso-Coello P, Schünemann HJ (2008). GRADE: an emerging consensus on rating quality of evidence and strength of recommendations. BMJ.

[28] Gupta P, Dadeya S, Kamlesh Kamlesh, Bhambhawani V (2017). Comparison of minimally invasive strabismus surgery (MISS) and conventional strabismus surgery using the limbal approach. J Pediatr Ophthalmol Strabismus.

[29] Summaiyya U, Amitava AK, Siddiqui Z (2025). A prospective, randomized parallel-design study to compare two conjunctival approaches in horizontal strabismus surgery: forniceal versus single-snip paralimbal. Indian J Ophthalmol.

[30] Froelich S, Viestenz A, Bredehorn-Mayr T (2021). Comparison of two conjunctival incision techniques in strabismus operations: analysis of the patient population in 2008. Klin Monbl Augenheilkd.

[31] Pellanda N, Mojon DS (2010). Combined horizontal rectus muscle minimally invasive strabismus surgery for exotropia. Can J Ophthalmol.

[32] Merino P, Blanco Domínguez I, Gómez de Liaño P (2016). Outcomes of minimally invasive strabismus surgery for horizontal deviation. Arch Soc Esp Oftalmol.

[33] Koranyi G, Seregard S, Kopp ED (2005). The cut-and-paste method for primary pterygium surgery: long-term follow-up. Acta Ophthalmol Scand.

[34] Abdallah M, Ali A, Shaarawy A, Masoud M (2024). Comparison between sutureless and sutured conjunctival autograft for surgical treatment of pterygium. Egypt J Hosp Med.

[35] Siddiqui MZ, Chauhan MZ, Stewart AF, Sallam AB (2022). Analysis of operational efficiency and cost differences between local and general anesthesia for vitreoretinal surgery. Healthcare (Basel).

